# The Role of the OBSCN Gene in Cardiomyopathy: A Review of Molecular Mechanisms and Clinical Significance

**DOI:** 10.3390/jcdd13100530

**Published:** 2026-10-21

**Authors:** Jingxiang Chen, Xiaoyun Sun, Sidi Wu, Jinzhu Hu, Yeqing Zou, Kui Hong, Yang Shen

**Affiliations:** 1 Department of Medical Genetics, The Second Affiliated Hospital of Nanchang University, Nanchang 330006, China; chenjingxiang66@gmail.com (J.C.); sunxiaoyun0506@163.com (X.S.); sidi.wu1217@gmail.com (S.W.); zouyeqing3366@163.com (Y.Z.); hongkui88@163.com (K.H.); 2 Department of Cardiovascular Medicine, The Second Affiliated Hospital of Nanchang University, Nanchang 330006, China; hujinzhu1983@sina.com; 3 Jiangxi Province Key Laboratory of Molecular Medicine, The Second Affiliated Hospital of Nanchang University, Nanchang 330006, China

**Keywords:** OBSCN, obscurin, cardiomyopathy, variant interpretation

## Abstract

Cardiomyopathies are genetically heterogeneous myocardial diseases that can lead to heart failure, arrhythmias, and sudden cardiac death. *OBSCN* encodes obscurin, a giant sarcomere-associated protein involved in sarcomere organization, cytoskeletal coupling, calcium handling, and cell–cell adhesion signaling. This review synthesizes current evidence on *OBSCN* variants in cardiomyopathy, focusing on their spectrum, phenotypic associations, and potential pathogenic mechanisms. Curated variants were systematically interpreted according to the American College of Medical Genetics and Genomics (ACMG) and the Association for Molecular Pathology (AMP) guidelines. Putative truncating variants predominated among variants of uncertain significance, and hypertrophic cardiomyopathy was the most frequently reported phenotype. Co-occurring variants in established or candidate cardiomyopathy-associated genes were reported for some OBSCN variants, particularly missense variants. However, co-occurrence alone does not establish an independent pathogenic contribution, oligogenic inheritance, or a genetic modifier effect. Potential mechanisms include obscurin insufficiency, destabilization of sarcomeric scaffolding, impaired sarcoplasmic reticulum coupling, dysregulated calcium homeostasis, altered mechanotransduction, and defective intercellular adhesion. Overall, *OBSCN* is an emerging but incompletely characterized cardiomyopathy-associated gene, and truncating variants warrant further clinical and mechanistic investigation.

## 1. Introduction

Cardiomyopathies comprise a heterogeneous group of disorders characterized by structural and/or functional abnormalities of the myocardium and commonly lead to heart failure, malignant arrhythmias, and sudden cardiac death [1,2]. Their pathogenesis is complex and involves diverse inheritance patterns and environmental contributors. With the development of high-throughput sequencing technologies, the contribution of genetic factors to cardiomyopathy has been increasingly recognized [3,4]. Early genetic studies focused primarily on variants in genes encoding sarcomeric proteins. As research has advanced, the spectrum of disease-associated genes has expanded to include genes encoding cytoskeletal proteins and proteins involved in cell–cell junctions [5]. In recent years, genes encoding proteins involved in sarcomere structural regulation and cytoskeletal coupling, including *OBSCN*, have attracted increasing attention and are thought to contribute to the development and progression of cardiomyopathy [1,6]. *OBSCN* encodes obscurin, a giant and structurally complex sarcomere-associated protein involved in sarcomere organization, calcium regulation, and intracellular signaling [7,8,9,10,11,12].

**Figure 1:**
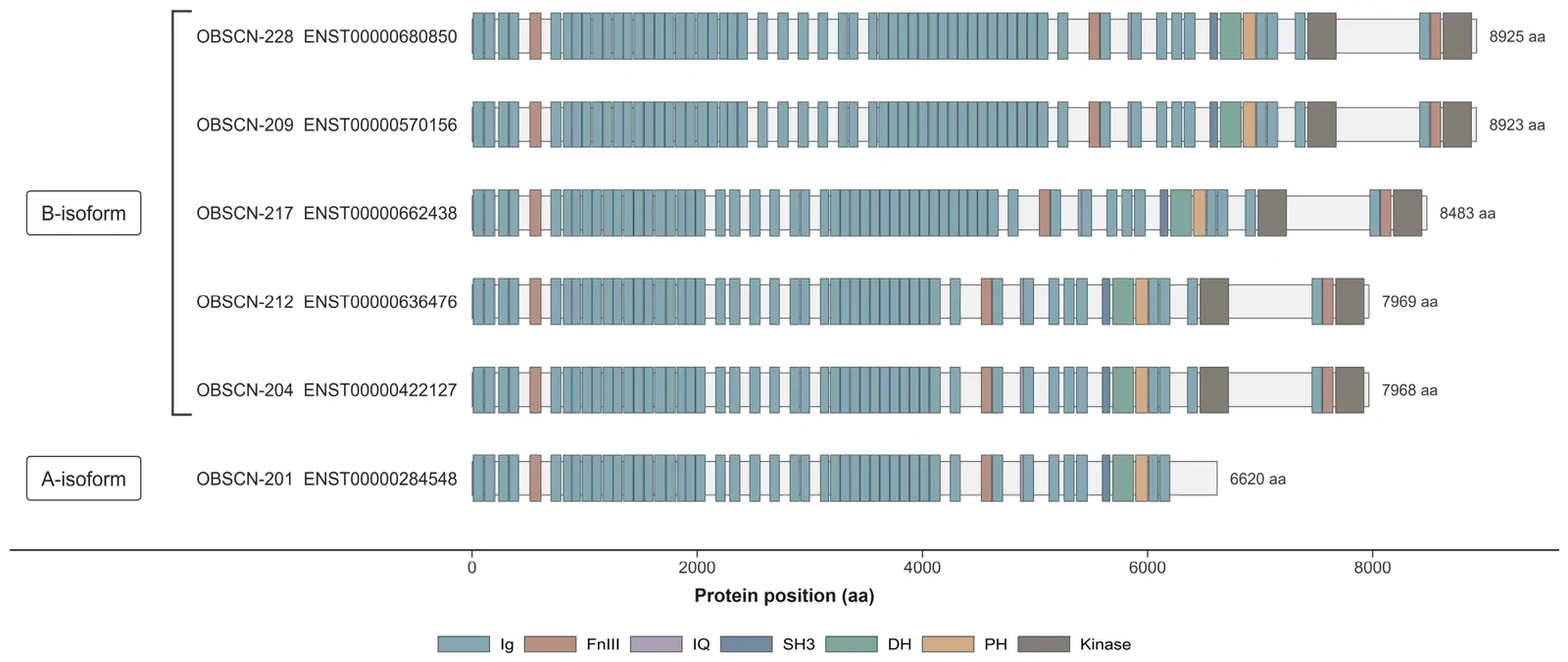
Major long isoforms of *OBSCN*.

It has been established that *OBSCN* undergoes extensive alternative splicing, generating multiple obscurin isoforms [13]. These obscurin isoforms differ in domain composition and tissue-specific expression patterns [12,14]. The combination of isoform diversity and obscurin’s highly modular architecture underlies its functional heterogeneity in striated muscle [13,14,15]. The major long *OBSCN* isoforms are obscurin-A and obscurin-B, as well as several short isoforms containing only kinase domains. The two long isoforms share an N-terminal structure composed of multiple tandem immunoglobulin-like (Ig) domains, followed by an IQ domain, and an SH3-RhoGEF-PH domain. Obscurin-A contains a non-modular C-terminal region with ankyrin-binding domains (ABDs), while obscurin-B contains two serine/threonine kinase domains, kin1 and kin2 (Figure 1) [13,16]. Notably, neither long isoform represents a single fixed protein species; each isoform may contain a variable number of Ig domains, while the overall functional domain organization is preserved [16].

In the heart, obscurin is widely distributed across multiple structures within the epicardium and myocardium, including the sarcolemma, intercalated discs, and sarcomeric striations, and both A and B isoforms are present in these sites [16]. Through its Ig1 domain, the N terminus of obscurin binds the M10 domain of titin, while its Ig3 domain interacts with myomesin, thereby enabling localization and stable integration of obscurin at the sarcomeric M-band through the titin–myomesin network [17,18]. The Ig58/59 domain of obscurin interacts with the ZIg9/10 region of titin; binding is primarily driven by ZIg9 and stabilized by ZIg10 [19]. Ig58/59 can also interact with a specific sequence in the titin splice isoform novex-3, thereby coupling obscurin to the Z-disc-associated sarcomeric cytoskeletal network [20,21]. In addition, Ig58/59 binds phospholamban (PLN), linking obscurin to the Ca^2+^-regulatory machinery of the sarcoplasmic reticulum (SR) and helping maintain Z-disc-associated cytoskeletal homeostasis and Ca^2+^ homeostasis in cardiomyocytes [20,21,22]. The internal IQ motif and tandem Dbl homology–pleckstrin homology (DH–PH) RhoGEF module of obscurin are thought to regulate cytoskeletal organization and myofibrillogenesis, rather than to mediate a reliably established canonical Rho small GTPase guanine-nucleotide exchange activity [23,24,25,26]. The C terminus of obscurin-A directly binds the C-terminal regulatory domain of ankyrin-B through its ankyrin-binding domains, mediates ankyrin-B localization at the sarcomeric M-line, and further recruits the PP2A complex to this region, thereby contributing to local signaling regulation and protein localization at the cardiomyocyte M-line [27]. The kinase domains of obscurin-B, kin1 and kin2, can localize to cell–cell junctions (intercalated discs) and directly phosphorylate the adhesion molecule N-cadherin, thereby regulating its binding partners, such as p120-catenin, as well as its stability and mechanical properties, ultimately influencing cardiomyocyte adhesion and mechanochemical coupling [28,29].

Previous studies have reported that *OBSCN* variants are associated with various cardiomyopathy phenotypes [12,30,31,32,33,34,35,36,37,38,39,40,41,42,43,44,45,46,47,48,49,50]. However, evidence supporting its pathogenic role remains limited, with existing research generally characterized by small sample sizes, a lack of validation in large-scale cohorts, and insufficient functional evidence [41,51,52,53,54]. This review integrates current evidence on OBSCN and cardiomyopathy, with a focus on gene–phenotype associations, variant distribution patterns, and potential pathogenic mechanisms, to inform future research and clinical interpretation.

## 2. Variant Screening and Genetic Evaluation of OBSCN

Reported *OBSCN* variants in patients with cardiomyopathy were identified through searches of PubMed, Europe PMC, and the Leiden Open Variation Database (LOVD), covering records from database inception through 1 September 2026. PubMed and Europe PMC searches were performed on 1 September 2026, and LOVD records were screened on the same date. Eligible studies provided specific *OBSCN* variant information from patients with a documented cardiomyopathy diagnosis. Reviews, studies limited to functional or animal experiments, and studies without relevant patient-level variant data were excluded. Duplicate records were resolved after transcript harmonization and HGVS normalization, and the same nucleotide variant was counted once while retaining all reported phenotypes, sources, and evidence. Detailed search strategies and study-selection procedures are provided in Supplementary File S1, Figure S1 and Tables S1–S3.

## 3. Spectrum and Distribution of OBSCN Variants

**Figure 2:**
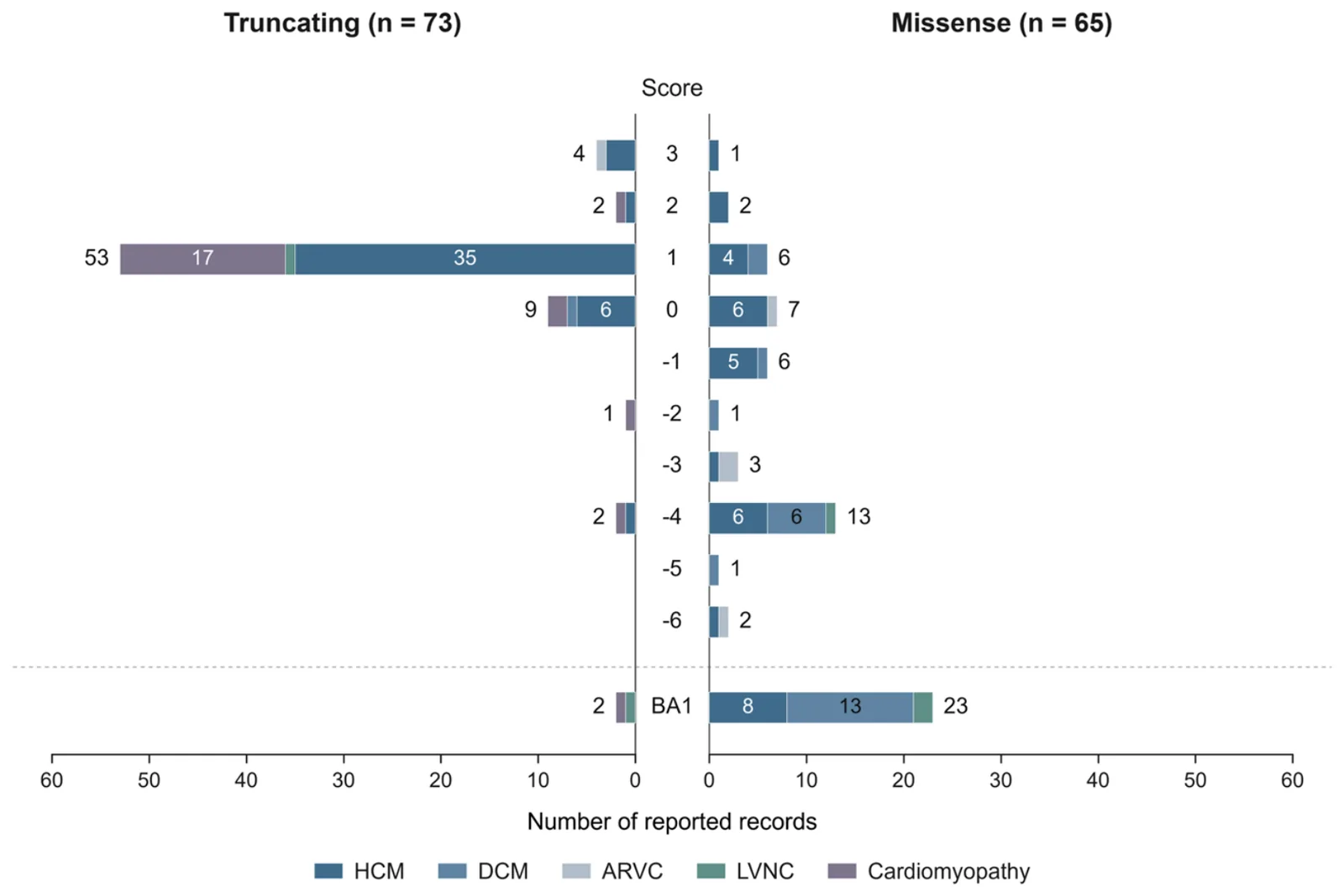
Distribution of cardiomyopathy phenotypes and variant classes among OBSCN variants. The horizontal dashed line separates the BA1 category from the numerical score categories.

A total of 138 distinct *OBSCN* variants were curated from cardiomyopathy patients, comprising 47 frameshift (34.1%), 20 nonsense (14.5%), 65 missense (47.1%), and 6 canonical splice-site variants (4.3%) (Table 1). Frameshift, nonsense, and canonical splice-site variants were grouped as putative truncating variants, together accounting for 73 out of 138 variants (52.9%). Using mutually exclusive phenotype categories, HCM was the most frequently associated phenotype (80/138, 58.0%), followed by DCM (25/138, 18.1%) and cardiomyopathy not otherwise specified (21/138, 15.2%). LVNC and ARVC each accounted for 5 variants (5/138, 3.6%) (Figure 2).

One additional variant was reported in both HCM and DCM patients (1/138, 0.7%). Another was reported in both DCM and LVNC patients (1/138, 0.7%).

All nonsense variants were classified as VUS, as were most frameshift variants (44/47, 93.6%). Missense variants were predominantly classified as B/LB (49/65, 75.4%), with the remaining 16 classified as VUS (24.6%). Among the 84 VUS variants, HCM remained the most frequently associated phenotype (58/84, 69.0%), followed by cardiomyopathy not otherwise specified (20/84, 23.8%). DCM accounted for 3 variants (3.6%), followed by ARVC (2/84, 2.4%) and LVNC (1/84, 1.2%). Putative truncating variants accounted for 68 out of 84 VUS variants (81.0%), including 45 out of 58 HCM-associated VUS variants (77.6%) (Figure 2). These proportions represent variant-level distributions within the curated dataset and should not be interpreted as phenotype-specific prevalence estimates of *OBSCN* variants.

Transcript-level analysis showed that 138 variants were present in four common long *OBSCN* transcripts. Of the 138 curated variants, 136 were present in the MANE Select transcript, while only two were absent, suggesting that most variants may affect major long isoforms. Notably, 46 *OBSCN* variants (33.3%) involved co-occurring variants in sarcomeric or other cardiomyopathy-associated genes. Co-occurrence was most frequently observed in HCM-associated variants (*n* = 36), followed by DCM (*n* = 6), ARVC (*n* = 2), and LVNC (*n* = 2). By *OBSCN* variant type, co-occurrence was most common among missense variants (*n* = 29), followed by frameshift variants (*n* = 11) and nonsense variants (*n* = 6). When stratified by *OBSCN* variant classification, co-occurring sarcomeric or cardiomyopathy-related variants were identified in 20 B/LB variants and 26 VUS variants (Table 1).

Several recurrent or linked variants required cautious interpretation. A5441T and R5301Q were reported to be in linkage disequilibrium. Although R5301Q was initially reported to affect obscurin–titin binding, enhance phospholamban binding, disrupt Ca^2+^ homeostasis, and contribute to electrophysiological abnormalities and myocardial remodeling, subsequent studies, together with its population frequency in gnomAD, do not support its classification as a highly penetrant pathogenic variant [22,55,56]. P2389Q was detected in three patients, two of whom carried additional sarcomeric gene variants, whereas one carried P2389Q alone [37]. P4171L was identified in two patients, both of whom carried the same sarcomeric variant, raising the possibility of linkage or a shared genetic background [37]. C4479Wfs*38 was detected in two patients, and *OBSCN* (NM_052843.4):c.19185_19188del was identified in three patients [38,41].

**Figure 3:**
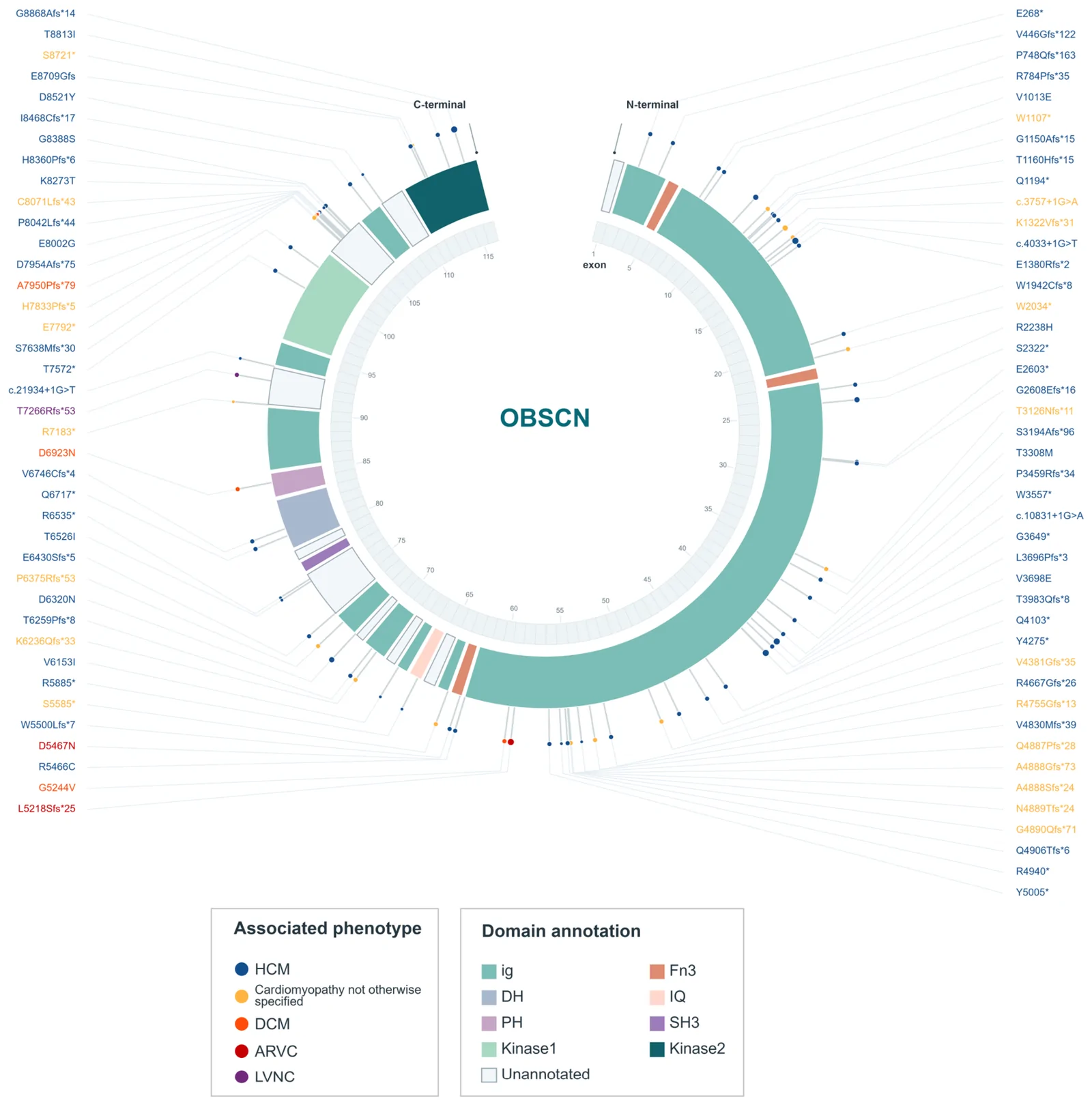
Exon-level and domain-level distribution of VUS OBSCN variants. The lollipop plot shows the distribution of MANE Select–mappable VUS variants across exons and corresponding obscurin protein domains. Dot colors indicate associated cardiomyopathy phenotypes, and domain colors indicate annotated protein domains. For visualization, introns were compressed, exons were colored according to domain annotation, and variants were colored according to cardiomyopathy subtype.

To minimize the influence of B/LB variants on analyses, only the 82 MANE Select–mappable VUS were mapped to the full-length MANE Select transcript. These variants were broadly distributed across exons 2–116 and included canonical splice-site variants in introns 12, 13, 40 and 93. The exon-domain lollipop plot showed that variants spanned multiple functional regions, including the Ig-rich region, central regulatory domains, and C-terminal kinase-containing regions, without clear restriction to a single protein domain or cardiomyopathy subtype (Figure 3). Among the MANE Select–mappable VUS, exon 105 contained the largest number of variants (*n* = 10), followed by exon 55 (*n* = 6). Exons 11 and 41 each contained three variants, whereas exons 7, 28, 56, 59, 62, 77, and 81 each contained two variants. All six variants included in the analysis from exon 55 were frameshift variants and were classified as VUS. Although exon 105 contained the largest number of variants, it included heterogeneous variant types, comprising six frameshift, one nonsense, and three missense variants. Because exon 105 is 2059 bp long and represents the largest exon in the MANE Select transcript, its apparent enrichment may partly reflect exon length. In contrast, exon 55 is only 267 bp long, suggesting possible local enrichment in exon 55. Overall, *OBSCN* variants showed broad distribution with local clustering; however, the existence of true mutational hotspots requires validation in larger cohorts with adjustment for exon length, transcript structure, sequencing coverage, and case ascertainment.

## 4. Pathogenic Implications and Interpretative Challenges of OBSCN Variants

In this review, we systematically summarized the spectrum of *OBSCN* variants reported in patients with cardiomyopathy. Of the 138 curated variants, 73 were putative truncating variants, including frameshift, nonsense, and canonical splice-site variants. Among these, 68 (93.2%) were classified as VUS. In contrast, 49 out of 65 missense variants (75.4%) were classified as benign or likely benign (B/LB). HCM was the most frequently associated phenotype in the curated dataset. The large proportion of unresolved truncating variants, together with their potential to disrupt protein function, supports further investigation of this class. However, these classifications reflect the available evidence and the research-based assessment framework used in this review. Neither pathogenicity nor penetrance can be established from this distribution alone.

The classification differences between truncating and missense variants may reflect their molecular consequences and the characteristics of the available evidence. First, OBSCN encodes obscurin, one of the largest human proteins, and, like TTN, undergoes extensive alternative splicing [7,13,57]. The interpretation of OBSCN variants may therefore involve some of the transcript-dependent considerations established for TTN. The consequences of missense variants generally depend on the specific amino acid substitution and the affected functional region. These transcript-dependent considerations highlight the need to investigate the actual molecular consequences of OBSCN truncating variants. Second, direct functional studies, family segregation analyses, and large-scale cohort data remain insufficient to establish the pathogenic contribution of individual OBSCN variants. Certain missense variants have functional or case-level evidence that warrants individual assessment despite the predominance of B/LB classifications within this class. Higher aggregate evidence scores require scrutiny of the supporting criteria, evidence strength, and conflicting findings. Furthermore, PVS1 requires an established loss-of-function disease mechanism and transcript-specific assessment; it was not applied in this review, and final classifications in the pathogenic direction were capped at VUS. Thus, the current findings support investigating truncating variants while retaining attention to missense variants with additional supporting evidence.

Truncating variants can substantially affect gene expression or protein function. Nevertheless, five putative truncating variants in this review met the BA1 or BS1 criteria and were classified as B/LB, indicating population frequencies inconsistent with a highly penetrant pathogenic role under the thresholds adopted in this study. These comprised two canonical splice-site variants and three frameshift variants. Canonical splice-site disruption may produce exon skipping without altering the reading frame, illustrating why a splice-site annotation does not necessarily indicate loss of function. For the frameshift variants, cardiac exon expression, transcript usage, and the retained functional regions provide potential explanations for their apparent tolerance. Extensive alternative splicing of OBSCN supports examining these consequences at the transcript level [13]. In particular, the myocardial expression and inclusion of the exon harboring C4479Wfs*38 may influence its functional impact, consistent with the importance of expression-aware variant interpretation [58]. Similar transcript-dependent effects have been reported for TTN [59]. Alternative obscurin isoforms could also modify the consequences of individual variants, although functional compensation remains to be demonstrated. These exceptions highlight the need to assess truncating variants in the context of exon expression, transcript usage, and the affected functional regions.

The potential role of OBSCN missense variants as genetic modifiers also warrants investigation. Of the 65 missense variants included in this review, 29 had reported co-occurring variants in other cardiomyopathy-associated genes. No such co-occurrence was reported for the remaining 36 variants. A5441T and R5301Q were reported as linked variants, further complicating attribution of an effect to either variant alone [33]. The frequent co-occurrence with variants in other genes, together with the high proportion of B/LB classifications, raises questions about whether many OBSCN missense variants are independently pathogenic. Some may represent incidental findings, whereas others could act as modifiers within specific genetic backgrounds, influencing cardiomyopathy susceptibility and phenotypic heterogeneity. However, co-occurrence alone does not establish a modifier effect or exclude an independent contribution from individual missense variants. Variants with supporting functional or clinical evidence therefore warrant targeted assessment to distinguish independent effects, modifier effects, and incidental co-occurrence. These co-occurrence counts describe distinct variant entries rather than independent patients.

Regarding the distribution of OBSCN variants across cardiomyopathy subtypes, HCM-associated variants were more numerous than those associated with other subtypes, but this finding requires cautious interpretation. Larger studies contributing to the OBSCN literature have included HCM-focused or unselected cardiomyopathy cohorts, potentially increasing the representation of HCM-associated variants in the curated dataset [38,41]. The greater number of HCM-associated variants therefore provides a substantial basis for further investigation in this phenotype, but does not directly establish greater disease specificity for HCM. Moreover, counts of distinct variants should be distinguished from patient-level prevalence when comparing cardiomyopathy subtypes.

According to the ClinGen gene–disease validity curations considered in this review, the associations of OBSCN with HCM and DCM are both classified as Limited. No formal ClinGen-rated association with ARVC or LVNC was identified in these curations. Accordingly, plausible mechanisms for individual truncating variants or functional abnormalities associated with certain missense variants provide research leads, but do not establish definitive relationships between OBSCN and these cardiomyopathy subtypes.

ClinGen gene–disease validity assessment relies on both genetic and experimental evidence. Genetic evidence includes case-level data, case–control studies, variant enrichment, and co-segregation in affected families; experimental evidence includes functional studies, model systems, and mechanistic investigations. Current OBSCN cardiomyopathy studies often address only one or a few of these domains. They have not yet established the convergent evidence required for a stronger gene–disease classification. In particular, the relationship between predicted truncating consequences and actual myocardial dysfunction remains insufficiently validated. Functional abnormalities associated with individual missense variants also require further integration with patient phenotypes and genetic evidence.

Overall, evidence for OBSCN-related cardiomyopathies remains limited but provides several directions for further investigation. Existing studies include case reports, patient cohorts, limited segregation data, and functional findings, while independent replication and consistent integration of genetic and experimental evidence remain insufficient. Truncating variants constitute a major unresolved group and warrant investigation of their actual transcript and protein consequences. Although missense variants were predominantly classified as B/LB, individual variants with additional supporting evidence remain worthy of study. Reports in ARVC and LVNC also justify further investigation, although the evidence is insufficient to establish definitive gene–disease relationships.

This review integrates genetic findings, variant characteristics, and functional evidence across cardiomyopathy phenotypes. It provides a basis for selecting truncating variants for further validation, evaluating missense variants with supporting evidence, and investigating possible contributions within broader genetic backgrounds. Such studies may help clarify the clinical significance of OBSCN variants and inform future gene–disease reassessment.

## 5. The Phenotypic Spectrum of OBSCN-Associated Cardiomyopathy

*OBSCN* variants have been identified in patients with diverse cardiomyopathy phenotypes. This section summarizes the clinical phenotypes reported in individuals carrying OBSCN variants. In unselected cardiomyopathy cohorts without strict phenotypic stratification, studies have reported a relative enrichment of OBSCN putative truncating variants, particularly among adults [38,41]. In several sequencing studies, *OBSCN* was among the most frequently implicated genes after *TTN* [32,34]. However, this observation varies across cohorts and may be influenced by gene size and background genetic variation. In HCM, some studies have associated truncating *OBSCN* variants with adverse clinical outcomes, although their relationship with disease severity remains unresolved [38]. Other reports have suggested a possible association with diastolic dysfunction, but these findings have not been consistently confirmed [41]. In DCM, *OBSCN* variants have been detected in patients with advanced disease, including cohorts of patients undergoing heart transplantation [31]. In some studies, their frequency appeared comparable to that of *TTN* variants, but these observations were based on relatively small sample sizes [31]. In both HCM and DCM cohorts, OBSCN variants often co-occurred with established sarcomeric gene variants [38,41]. However, co-occurrence alone does not establish whether OBSCN variants independently contribute to disease, act as modifiers, or represent incidental findings.

In addition to HCM and DCM, *OBSCN* variants have also been reported in other cardiomyopathy subtypes, including ARVC and LVNC [30,36,41]. However, current evidence is primarily limited to individual cases or small case series, often with additional co-occurring variants, and their pathogenic relevance remains unclear. Notably, in a cohort study of 325 patients with DCM and 10 with LVNC, *OBSCN* variants were detected in 3 patients with LVNC and 1 with DCM [30]. This finding suggests that *OBSCN* variants appeared more frequent in the LVNC subgroup in this dataset, but given the small sample size, this observation should be interpreted with caution.

## 6. Molecular Mechanisms, In Vitro Data, and Animal Models of OBSCN-Related Cardiomyopathy

Current evidence suggests that the pathogenic mechanisms underlying *OBSCN*-related cardiomyopathy are better conceptualized as a stepwise cascade of structural scaffold destabilization, aberrant signal transduction, and disrupted calcium homeostasis, rather than as a defect in a single molecular function. Obscurin isoforms encoded by *OBSCN* are incorporated into nascent myofibrils early during cardiac development. Obscurin contributes to sarcomere assembly, maturation, spatial organization, and remodeling. During development, its distribution gradually shifts from an early Z-band-predominant pattern to localization at the M-line [7,60,61,62]. Obscurin is also essential for the lateral alignment of striated myofibrils and for maintaining the stability of the contractile apparatus. Loss of obscurin can cause abnormal cardiac development, disorganized myofibrillar architecture, and impaired mechanical stability [23,63,64]. Thus, loss of obscurin may initially compromise sarcomeric organization.

At the level of molecular localization, proper anchoring of obscurin depends on its interactions with sarcomeric proteins such as titin and myomesin. Disruption of these interactions can lead to obscurin mislocalization and destabilization of M-line- and Z-disc-associated structural complexes. Disruption of these interactions has been implicated in inherited myopathies and cardiomyopathies [17,19,33]. Beyond its role as a sarcomeric component, obscurin functions as a central hub linking sarcomeres to the SR, the membrane cytoskeleton, and associated signaling complexes. This role contributes to structural coupling between nascent myofibrils and the sarcoplasmic reticulum and helps maintain the localization and function of M-line-associated macromolecular complexes [27,62,65]. These functions are mediated through the recruitment of ankyrin-B, sAnk1.5, PP2A, and related molecules [27,65]. Therefore, the structural changes or loss of obscurin caused by *OBSCN* variants may affect not only the sarcomere itself, but also the broader sarcomere–membrane–organelle coupling network [66,67].

Beyond its structural roles, obscurin contains multiple functional domains, including RhoGEF, MLCK, and kinase domains, that participate in small GTPase signaling, hypertrophic growth regulation, and cell adhesion-related pathways [13,14]. Aortic constriction models suggest that *OBSCN* is activated early after pressure overload; its upregulation is associated with nascent myofibrillogenesis and hypertrophic growth, and obscurin–MLCK may mediate mechanostress-induced signaling [23]. Zebrafish studies further suggest that the RhoGEF-containing region of obscurin is required for cardiac development, cardiomyocyte maturation, and intercalated disc formation [26]. Moreover, kinase domains of obscurin, especially obscurin-Kin1, can affect the p120-catenin/RhoA axis by regulating the phosphorylation of N-cadherin Ser788, thereby altering the strength of intercellular coupling between cardiomyocytes; in parallel, the smaller obscurin isoforms obscurin-40/80 localize to intercalated discs and regulate the PI3K/AKT/mTOR pathway through their PH domain, limiting cardiomyocyte hypertrophy and maintaining stable adhesion [28,29,68]. These results suggest that *OBSCN* abnormalities can also promote disease progression by disrupting adhesion- and growth-related signaling networks.

Accumulating evidence indicates that impaired calcium cycling and abnormal excitation–contraction coupling are central downstream components of *OBSCN*-related pathogenesis. Deletion of the Ig58/59 domain has been demonstrated to induce age-dependent ventricular remodeling and arrhythmias, accompanied by abnormal regulation of PLN, SERCA2, and RyR2 [20]. In atrial myocardium, Ig58/59 deletion can also disrupt Z-disc organization and the transverse-axial tubular network, leading to asynchronous Ca^2+^ release, increased SR Ca^2+^ leak, and Ca^2+^-dependent arrhythmogenesis [21,69]. Global loss of obscurin results in sarcoplasmic reticulum structural disorganization, dysregulated SERCA/PLB signaling, impaired Ca^2+^ reuptake, and reduced reserve under β-adrenergic stress, thereby causing compensatory dilated cardiomyopathy and increased susceptibility to arrhythmias [66]. Cardiac-specific combined deletion of Obsc and Obsl1 caused diastolic dysfunction accompanied by abnormalities in the sarcoplasmic reticulum architecture, calcium cycling, mitochondrial structure, metabolism, and mitophagy, supporting partly redundant cardiac functions of the two proteins [67]. In human-induced pluripotent stem cell-derived cardiomyocytes, *OBSCN* variants can reduce obscurin and Ank1.5 protein abundance and disrupt their localization, accompanied by increased L-type Ca^2+^ current, Z-line ultrastructural abnormalities, and lipid accumulation. These findings suggest that *OBSCN* variants may simultaneously drive electrophysiological abnormalities and metabolic reprogramming [36]. Furthermore, proteomic studies further indicate that certain pathogenic *OBSCN* variants induce systemic changes in proteins related to calcium handling, ion channels, and the membrane cytoskeleton [56]. Notably, not all reported protein interactions have been sufficiently validated as pathogenic in vivo. For instance, one study proposed that the interaction between obscurin and PLN may primarily depend on exposure of the PLN transmembrane region under in vitro conditions [55]. Therefore, the physiological and pathogenic relevance of this interaction in vivo remains uncertain.

To date, several functional studies have investigated *OBSCN* variants across different cardiomyopathy subtypes, including in vitro assays of *OBSCN* variants, variant-specific mammalian knock-in models, and splicing assays using cardiac tissue from individuals carrying *OBSCN* variants (Table 2). Some OBSCN variants may cause haploinsufficiency while largely preserving protein localization [31]. This observation suggests that distinct classes of *OBSCN* variants may cause disease through different mechanisms, including protein insufficiency, mislocalization, impaired molecular interactions, or defective signaling [31,33,36]. Overall, disruptions of obscurin-mediated sarcomeric scaffolding, organelle positioning, cell-junction complexes, and Ca^2+^ regulatory networks appear to contribute to OBSCN-related cardiomyopathy. These abnormalities may ultimately contribute to myocardial hypertrophy, dilation, diastolic dysfunction, and arrhythmias.

## 7. Limitations and Future Perspectives

This review has several limitations. First, the variants included in this review were interpreted using published literature, database annotations, and ACMG/AMP criteria, which may introduce selection and information bias. The 2015 ACMG/AMP guidelines provide a framework for clinical variant interpretation, but their application to OBSCN requires caution because its gene–disease validity in cardiomyopathy remains limited [70]. Several evidence categories, including population frequency, segregation, functional studies, and case enrichment, were limited by the available data or the absence of OBSCN-specific specifications. In particular, the population-frequency thresholds adopted from other cardiomyopathy genes have not been calibrated for OBSCN. PVS1 was not applied, and final classifications in the pathogenic direction were capped at VUS because of the limited gene–disease validity. This classification boundary should be distinguished from the limitations of the study-specific evidence assessment. The use of thresholds and specifications extrapolated from other genes introduces uncertainty, particularly in frequency-dependent B/LB classifications. These findings therefore require cautious interpretation pending the validation of OBSCN-specific thresholds and interpretation rules. The actual effects of individual missense and canonical splice-site variants on obscurin structure, sarcomere stability, and cardiomyocyte function remain incompletely characterized. Although most missense variants were classified as B/LB, 16 remained VUS and require further assessment.

Second, some cases lacked complete family co-segregation data and follow-up, limiting the assessment of penetrance and genotype–phenotype correlations and restricting the application of PP1 evidence. At the population level, cohort evidence was uneven across cardiomyopathy subtypes and variant classes, limiting the assessment of case enrichment and the application of PS4. Variant-specific functional evidence also remained insufficient for many variants, restricting the application of PS3. Gene-level knockout or domain-deletion experiments provide biological context but do not directly establish the effects of individual human variants. Region- and transcript-dependent effects are well-established for TTN, highlighting the importance of investigating these factors in OBSCN [71,72]. Without systematic transcript-aware assessment, the molecular consequences and potential disease contribution of some OBSCN truncating variants may be overestimated or underestimated. Finally, molecular studies of OBSCN-related cardiomyopathy remain relatively limited, and the links between individual variants, altered obscurin function, and myocardial disease have not been fully defined.

Future studies should develop and validate OBSCN-specific interpretation rules within the ACMG/AMP and ClinGen SVI frameworks, with particular attention to cardiac transcript usage and gene-specific disease mechanisms. For missense variants, additional genetic and functional studies are required to clarify their functional and clinical significance. Systematic functional studies, including RNA splicing assays, induced pluripotent stem cell-derived cardiomyocyte models, CRISPR/Cas9 editing models, and protein interaction analyses, are needed to determine how different variant types affect obscurin protein stability, sarcomere structure, and signal transduction. Integrating these findings with family segregation and independent cohort data will be essential for assessing their relevance to cardiomyopathy.

## 8. Conclusions

Current evidence suggests that OBSCN may contribute to cardiomyopathy through multiple molecular mechanisms. Truncating variants represent important candidates for further pathogenicity assessment, although their effects may depend on transcript context, exon expression, domain location, and nonsense-mediated decay. The region- and transcript-dependent effects described for TTN provide a useful framework for investigating these factors in OBSCN. Our review highlighted the frequent co-occurrence of OBSCN missense variants with variants in established or candidate cardiomyopathy-associated genes. This raises the possibility that some OBSCN variants could influence disease susceptibility or phenotypic expression within a broader genetic background. However, they may also represent incidental background variation, and the available evidence does not establish oligogenic inheritance or a genetic modifier effect. Future studies integrating transcript-aware variant interpretation, multi-omics approaches, long-read sequencing, and functional models will be important for clarifying variant effects and genotype–phenotype relationships. In addition, larger multicenter cohorts with standardized clinical phenotyping and long-term follow-up are needed to assess penetrance and determine whether specific OBSCN variants are associated with prognosis or arrhythmic risk in cardiomyopathy.

## Figures and Tables

**Table 1:** Summary of OBSCN variants reported in patients with cardiomyopathy.

hg38	Transcript	cDNA	Protein	Protein (HGVS)	Exon	Variant Type	Phenotype	ACMG_Criteria	Score	ACMG/AMP Classification	Co-Occurring Cardiomyopathy Variant	References
chr1-228212585-G-T	NM_001386125.1	c.802G>T	E268*	p.(Glu268Ter)	exon 2	nonsense	HCM	PM2_Supporting	1	VUS	MYBPC3 p.E728* (not provide)	[34]
chr1-228213482-G-C	NM_001386125.1	c.1030G>C	V344L	p.(Val344Leu)	exon 3	missense	DCM	BA1	/	B	LMNA p.L140_A146dup (not provide)	[34]
chr1-228213629-A-G	NM_001386125.1	c.1177A>G	N393D	p.(Asn393Asp)	exon 3	missense	DCM	BP4_Moderate	−2	LB		[50]
chr1-228214250--G	NM_001386125.1	c.1336dup	V446Gfs*122	p.(Val446GlyfsTer122)	exon 4	frameshift	HCM	PM2_Supporting	1	VUS		[38]
chr1-228216468-G-A	NM_001386125.1	c.2143G>A	E715K	p.(Glu715Lys)	exon 7	missense	HCM	BA1	/	B	TNNT2 p.R102Q (not provide)	[34]
chr1-228216565-C-	NM_001386125.1	c.2243del	P748Qfs*163	p.(Pro748GlnfsTer163)	exon 7	frameshift	HCM	PM2_Supporting	1	VUS	JPH2 p.G199D (not provide)	[45]
chr1-228216674--C	NM_001386125.1	c.2350dup	R784Pfs*35	p.(Arg784ProfsTer35)	exon 7	frameshift	HCM	PM2_Supporting	1	VUS		[38]
chr1-228224567-T-A	NM_001386125.1	c.3038T>A	V1013E	p.(Val1013Glu)	exon 10	missense	HCM	PM2_Supporting, PP3	2	VUS		[43]
chr1-228224692-G-A	NM_001386125.1	c.3163G>A	E1055K	p.(Glu1055Lys)	exon 10	missense	DCM	BS1	−4	LB		[31]
chr1-228243298-G-A	NM_001386125.1	c.3321G>A	W1107*	p.(Trp1107Ter)	exon 11	nonsense	Cardiomyopathy	PM2_Supporting	1	VUS		[41]
chr1-228243423-G-	NM_001386125.1	c.3449del	G1150Afs*15	p.(Gly1150AlafsTer15)	exon 11	frameshift	HCM	PM2_Supporting	1	VUS	MYBPC3 p.L460fs (not provide)	[38]
chr1-228243454--A	NM_001386125.1	c.3476_3477insA	T1160Hfs*15	p.(Thr1160HisfsTer15)	exon 11	frameshift	HCM	PM2_Supporting	1	VUS		[38]
chr1-228244394-C-T	NM_001386125.1	c.3580C>T	Q1194*	p.(Gln1194Ter)	exon 12	nonsense	HCM	PM2_Supporting	1	VUS		[38]
chr1-228244407-C-A	NM_001386125.1	c.3593C>A	T1198K	p.(Thr1198Lys)	exon 12	missense	DCM	PM2_Supporting, BP4_Moderate	−1	LB	DSP p.A1274P (not provide)	[34]
chr1-228244476-G-A	NM_001386125.1	c.3662G>A	R1221Q	p.(Arg1221Gln)	exon 12	missense	HCM	BS1	−4	LB	MYOM1 p.S451Ffs*27 (VUS) MYH7 p.N1284 = (VUS)	[43]
chr1-228244572-G-A	NM_001386125.1	c.3757+1G>A	?	p.(?)	intron 12	canonical ± 1 or 2 splice sites	Cardiomyopathy	PM4	2	VUS		[41]
chr1-228245593-G-A	NM_001386125.1	c.3938G>A	R1313Q	p.(Arg1313Gln)	exon 13	missense	HCM	BS1	−4	LB	MYOM1 p.S451Ffs*27 (not provide)	[37]
chr1-228245615-GGGCAAAGCAGATGCTG-	NM_001386125.1	c.3963_3979del	K1322Vfs*31	p.(Lys1322ValfsTer31)	exon 13	frameshift	Cardiomyopathy	PM2_Supporting	1	VUS		[41]
chr1-228245689-G-T	NM_001386125.1	c.4033+1G>T	?	p.(?)	intron 13	canonical ± 1 or 2 splice sites	HCM	PM4, PM2_Supporting	3	VUS		[38]
chr1-228246631-A-	NM_001386125.1	c.4137del	E1380Rfs*2	p.(Glu1380ArgfsTer2)	exon 14	frameshift	HCM	PM2_Supporting	1	VUS		[38]
chr1-228268697-G-A	NM_001386125.1	c.5581G>A	V1861M	p.(Val1861Met)	exon 19	missense	HCM	BS1, BP4_Moderate	−6	LB	NEXN p.I467del (not provide)	[34]
chr1-228272060-G-	NM_001386125.1	c.5826delG	W1942Cfs*8	p.(Trp1942CysfsTer8)	exon 20	frameshift	HCM	PM2_Supporting	1	VUS		[38]
chr1-228273387-G-A	NM_001386125.1	c.6102G>A	W2034*	p.(Trp2034Ter)	exon 21	nonsense	Cardiomyopathy	PM2_Supporting	1	VUS		[41]
chr1-228274298-C-T	NM_001386125.1	c.6662C>T	T2221M	p.(Thr2221Met)	exon 23	missense	DCM	BA1	/	B		[49]
chr1-228274306-C-T	NM_001386125.1	c.6670C>T	R2224W	p.(Arg2224Trp)	exon 23	missense	DCM	BS1, BP4	−5	LB		[46]
chr1-228274349-G-A	NM_001386125.1	c.6713G>A	R2238H	p.(Arg2238His)	exon 23	missense	HCM	PS4_Supporting	1	VUS	CACNB2 p.W652C (not provide)	[45]
chr1-228274728-C-G	NM_001386125.1	c.6965C>G	S2322*	p.(Ser2322Ter)	exon 24	nonsense	HCM	PM2_Supporting, PS4_Supporting	2	VUS	MYBPC3 p.Q374* (not provide)	[38,45]
chr1-228275847-C-A	NM_001386125.1	c.7166C>A	P2389Q	p.(Pro2389Gln)	exon 25	missense	HCM	BA1	/	B	RYR2 p.G1752D (VUS)	[37,43]
chr1-228276711-T-A	NM_001386125.1	c.7607T>A	V2536D	p.(Val2536Asp)	exon 26	missense	DCM	BS1	−4	LB		[31]
chr1-228277241-G-T	NM_001386125.1	c.7807G>T	E2603*	p.(Glu2603Ter)	exon 28	nonsense	HCM	PM2_Supporting	1	VUS		[38]
chr1-228277255-G-	NM_001386125.1	c.7823delG	G2608Efs*16	p.(Gly2608GlufsTer16)	exon 28	frameshift	HCM	PM2_Supporting	1	VUS	MYH7 p.Q1206K (not provide)	[38]
chr1-228279394-T-C	NM_001386125.1	c.8633T>C	V2878A	p.(Val2878Ala)	exon 32	missense	LVNC	BA1	/	B	PLEC p.T3796I (not provide)	[39]
chr1-228280293-A-G	NM_001386125.1	c.9065A>G	N3022S	p.(Asn3022Ser)	exon 34	missense	HCM	BA1	/	B		[43]
chr1-228280688--A	NM_001386125.1	c.9376dup	T3126Nfs*11	p.(Thr3126AsnfsTer11)	exon 35	frameshift	Cardiomyopathy	PM2_Supporting	1	VUS		[41]
chr1-228282026-C-	NM_001386125.1	c.9579del	S3194Afs*96	p.(Ser3194AlafsTer96)	exon 36	frameshift	HCM	PM2_Supporting	1	VUS		[45]
chr1-228283183-C-T	NM_001386125.1	c.9923C>T	T3308M	p.(Thr3308Met)	exon 37	missense	HCM	PM2_Supporting	1	VUS	MYH7 p.Q1267H (VUS)	[43]
chr1-228286159-C-	NM_001386125.1	c.10376del	P3459Rfs*34	p.(Pro3459ArgfsTer34)	exon 39	frameshift	HCM	PM2_Supporting	1	VUS		[45]
chr1-228286879-G-A	NM_001386125.1	c.10671G>A	W3557*	p.(Trp3557Ter)	exon 40	nonsense	HCM	PM2_Supporting	1	VUS	MYH7 p.A797T (not provide)	[38]
chr1-228287040-G-A	NM_001386125.1	c.10831+1G>A	?	p.(?)	intron 40	canonical ± 1 or 2 splice sites	HCM	PM4, PM2_Supporting	3	VUS		[38]
chr1-228287807-G-T	NM_001386125.1	c.10945G>T	G3649*	p.(Gly3649Ter)	exon 41	nonsense	HCM	PM2_Supporting	1	VUS		[38]
chr1-228287949-T-	NM_001386125.1	c.11087del	L3696Pfs*3	p.(Leu3696ProfsTer3)	exon 41	frameshift	HCM	PM2_Supporting	1	VUS		[38]
chr1-228287955-T-A	NM_001386125.1	c.11093T>A	V3698E	p.(Val3698Glu)	exon 41	missense	HCM	PM2_Supporting, PP3_Moderate	3	VUS		[43]
chr1-228292577-C-	NM_001386125.1	c.11946del	T3983Qfs*8	p.(Thr3983GlnfsTer8)	exon 45	frameshift	HCM	PM2_Supporting	1	VUS	MYH6 p.E1858G (not provide)	[45]
chr1-228292618-G-C	NM_001386125.1	c.11986G>C	V3996L	p.(Val3996Leu)	exon 45	missense	DCM	BA1	/	B	TTN c.39893-1G>A (not provide) TTN p.K3240R (not provide)	[42]
chr1-228293505-C-T	NM_001386125.1	c.12307C>T	Q4103*	p.(Gln4103Ter)	exon 46	nonsense	HCM	PM2_Supporting	1	VUS		[38]
chr1-228294239-C-T	NM_001386125.1	c.12506C>T	A4169V	p.(Ala4169Val)	exon 47	missense	ARVC	PM2_Supporting, BP4_Strong	−3	LB		[34]
chr1-228294245-C-T	NM_001386125.1	c.12512C>T	P4171L	p.(Pro4171Leu)	exon 47	missense	HCM	BS1, PS4_Supporting	−3	LB	MYH7 p.T1377M (LP)	[37,43]
chr1-228294922-C-G	NM_001386125.1	c.12825C>G	Y4275*	p.(Tyr4275Ter)	exon 48	nonsense	HCM	PM2_Supporting	1	VUS		[38]
chr1-228298647-TG-	NM_001386125.1	c.13142_13143del	V4381Gfs*35	p.(Val4381GlyfsTer35)	exon 49	frameshift	Cardiomyopathy	PM2_Supporting	1	VUS		[41]
chr1-228299476-AT-	NM_001386125.1	c.13428_13429delAT	C4479Wfs*38	p.(Cys4479TrpfsTer38)	exon 50	frameshift	HCM&DCM	BS1	−4	LB		[41]
chr1-228303935-AG-	NM_001386125.1	c.13999_14000delAG	R4667Gfs*26	p.(Arg4667GlyfsTer26)	exon 52	frameshift	HCM	PM2_Supporting	1	VUS	MYBPC3 p.R845fs (not provide)	[38]
chr1-228304563-AG-	NM_001386125.1	c.14263_14264del	R4755Gfs*13	p.(Arg4755GlyfsTer13)	exon 53	frameshift	Cardiomyopathy	PM2_Supporting	1	VUS		[41]
chr1-228305340-AT-	NM_001386125.1	c.14484_14485delAT	V4830Mfs*39	p.(Val4830MetfsTer39)	exon 54	frameshift	HCM	/	0	VUS		[38]
chr1-228306496--GCTCCCTTGG	NM_001386125.1	c.14657_14658insCCCTTGGGCT	Q4887Pfs*28	p.(Gln4887ProfsTer28)	exon 55	frameshift	Cardiomyopathy	PM2_Supporting	1	VUS		[41]
chr1-228306502--A	NM_001386125.1	c.14661_14662insA	A4888Sfs*24	p.(Ala4888SerfsTer24)	exon 55	frameshift	Cardiomyopathy	PM2_Supporting	1	VUS		[41]
chr1-228306502--GG	NM_001386125.1	c.14661_14662dup	A4888Gfs*73	p.(Ala4888GlyfsTer73)	exon 55	frameshift	Cardiomyopathy	PM2_Supporting	1	VUS		[41]
chr1-228306507--CGGC	NM_001386125.1	c.14665_14666insCGGC	N4889Tfs*24	p.(Asn4889ThrfsTer24)	exon 55	frameshift	Cardiomyopathy	PM2_Supporting	1	VUS		[41]
chr1-228306509--CA	NM_001386125.1	c.14667_14668insCA	G4890Qfs*71	p.(Gly4890GlnfsTer71)	exon 55	frameshift	Cardiomyopathy	PM2_Supporting	1	VUS		[41]
chr1-228306515-C-T	NM_001386125.1	c.14674C>T	R4892W	p.(Arg4892Trp)	exon 55	missense	DCM	BA1	/	B		[49]
chr1-228306556--A	NM_001386125.1	c.14715dupA	Q4906Tfs*6	p.(Gln4906ThrfsTer6)	exon 55	frameshift	HCM	PM2_Supporting	1	VUS	MYBPC3 p.E1050K (not provide)	[38]
chr1-228306906-C-A	NM_001386125.1	c.14803C>A	P4935T	p.(Pro4935Thr)	exon 56	missense	LVNC	BA1	/	B	MYH7 p.P161T (not provide)	[39]
chr1-228306921-C-T	NM_001386125.1	c.14818C>T	R4940*	p.(Arg4940Ter)	exon 56	nonsense	HCM	/	0	VUS	MYH7 p.R403Q (not provide)	[38]
chr1-228307118-C-G	NM_001386125.1	c.15015C>G	Y5005*	p.(Tyr5005Ter)	exon 56	nonsense	HCM	PM2_Supporting	1	VUS		[38]
chr1-228308282-G-A	NM_001386125.1	c.15509G>A	R5170H	p.(Arg5170His)	exon 58	missense	HCM	BA1	/	B	MYBPC3 c.506-2A>C (not provide)	[34]
chr1-228308312-C-T	NM_001386125.1	c.15539C>T	T5180M	p.(Thr5180Met)	exon 58	missense	DCM	BS1	−4	LB		[34]
chr1-228308320-G-T	NM_001386125.1	c.15547G>T	D5183Y	p.(Asp5183Tyr)	exon 58	missense	HCM	BA1	/	B		[37,43]
chr1-228309140-C-	NM_001386125.1	c.15652del	L5218Sfs*25	p.(Leu5218SerfsTer25)	exon 59	frameshift	ARVC	PS2_Moderate, PM2_Supporting	3	VUS	MYBPC3 p.M1I (not provide)	[36]
chr1-228309219-G-T	NM_001386125.1	c.15731G>T	G5244V	p.(Gly5244Val)	exon 59	missense	DCM	PM2_Supporting	1	VUS		[35]
chr1-228315865-G-A	NM_001386125.1	c.15902G>A	R5301Q	p.(Arg5301Gln)	exon 61	missense	HCM	BA1	/	B		[33]
chr1-228316873-G-A	NM_001386125.1	c.16321G>A	A5441T	p.(Ala5441Thr)	exon 62	missense	HCM	BA1	/	B		[33]
chr1-228316898-T-G	NM_001386125.1	c.16346T>G	L5449R	p.(Leu5449Arg)	exon 62	missense	HCM	BS1	−4	LB		[48]
chr1-228316948-C-T	NM_001386125.1	c.16396C>T	R5466C	p.(Arg5466Cys)	exon 62	missense	HCM	PS4_Supporting	1	VUS	MYBPC3 p.C1124* (not provide)	[45]
chr1-228316951-G-A	NM_001386125.1	c.16399G>A	D5467N	p.(Asp5467Asn)	exon 62	missense	ARVC	/	0	VUS	DSG2 p.F531C (not provide)	[44]
chr1-228317528--T	NM_001386125.1	c.16498dup	W5500Lfs*7	p.(Trp5500LeufsTer7)	exon 63	frameshift	HCM	PM2_Supporting	1	VUS		[38]
chr1-228317556-G-A	NM_001386125.1	c.16525G>A	G5509S	p.(Gly5509Ser)	exon 63	missense	DCM	BA1	/	B		[34,49]
chr1-228317599-C-A	NM_001386125.1	c.16568C>A	T5523K	p.(Thr5523Lys)	exon 63	missense	HCM	PM2_Supporting, BP4_Moderate	−1	LB		[45]
chr1-228317722-C-T	NM_001386125.1	c.16691C>T	P5564L	p.(Pro5564Leu)	exon 63	missense	HCM	BA1	/	B		[43]
chr1-228317925-C-G	NM_001386125.1	c.16754C>G	S5585*	p.(Ser5585Ter)	exon 64	nonsense	Cardiomyopathy	PM2_Supporting	1	VUS		[41]
chr1-228318024-A-C	NM_001386125.1	c.16853A>C	E5618A	p.(Glu5618Ala)	exon 64	missense	DCM	BA1	/	B		[49]
chr1-228321377-C-G	NM_001386125.1	c.17407C>G	Q5803E	p.(Gln5803Glu)	exon 66	missense	HCM	PM2_Supporting, BP4_Moderate	−1	LB	MYH6 p.E1858G (not provide) TTN p.T22168I (not provide)	[45]
chr1-228321408-G-A	NM_001386125.1	c.17438G>A	R5813H	p.(Arg5813His)	exon 66	missense	DCM	BA1	/	B		[31]
chr1-228321623-C-T	NM_001386125.1	c.17653C>T	R5885*	p.(Arg5885Ter)	exon 66	nonsense	HCM	/	0	VUS	MYH7 p.R1420W (not provide)	[38]
chr1-228321878-C-T	NM_001386125.1	c.17908C>T	R5970W	p.(Arg5970Trp)	exon 66	missense	DCM	BS1	−4	LB		[46]
chr1-228323519-G-A	NM_001386125.1	c.18436G>A	V6146M	p.(Val6146Met)	exon 67	missense	DCM	BS1	−4	LB		[49]
chr1-228323540-G-A	NM_001386125.1	c.18457G>A	V6153I	p.(Val6153Ile)	exon 67	missense	HCM	BP4, PS4_Supporting	0	VUS	MYBPC3 p.E334K (LP) MYH7 p.R442C (LP)	[37,43]
chr1-228323598-G-A	NM_001386125.1	c.18515G>A	R6172H	p.(Arg6172His)	exon 67	missense	HCM	BS1	−4	LB		[32]
chr1-228333300--C	NM_001386125.1	c.18705dup	K6236Qfs*33	p.(Lys6236GlnfsTer33)	exon 69	frameshift	Cardiomyopathy	PM2_Supporting	1	VUS		[41]
chr1-228333630-A-	NM_001386125.1	c.18775del	T6259Pfs*8	p.(Thr6259ProfsTer8)	exon 70	frameshift	HCM	PM2_Supporting	1	VUS		[38]
chr1-228334544-G-A	NM_001386125.1	c.18958G>A	D6320N	p.(Asp6320Asn)	exon 71	missense	HCM	PM2_Supporting, PS4_Supporting	2	VUS		[45]
chr1-228335146-CT-	NM_001386125.1	c.19124_19125del	P6375Rfs*53	p.(Pro6375ArgfsTer53)	exon 73	frameshift	Cardiomyopathy	PM2_Supporting	1	VUS		[41]
chr1-228335801-GGAG-	NM_001386125.1	c.19288_19291delGAGG	E6430Sfs*5	p.(Glu6430SerfsTer5)	exon 74	frameshift	HCM	PM2_Supporting	1	VUS	MYBPC3 p.R977fs (not provide)	[38]
chr1-228337055-T-C	NM_001386125.1	c.19460T>C	V6487A	p.(Val6487Ala)	exon 76	missense	DCM	BA1	/	B		[49]
chr1-228337289-C-T	NM_001386125.1	c.19577C>T	T6526I	p.(Thr6526Ile)	exon 77	missense	HCM	/	0	VUS	MYH7 p.E1902Q (VUS)	[37,43]
chr1-228337315-C-T	NM_001386125.1	c.19603C>T	R6535*	p.(Arg6535Ter)	exon 77	nonsense	HCM	/	0	VUS		[38]
chr1-228338122-C-T	NM_001386125.1	c.19850C>T	A6617V	p.(Ala6617Val)	exon 78	missense	DCM	BA1	/	B	PKP2 p.I681fs (not provide)	[34]
chr1-228338877-T-A	NM_001386125.1	c.19980T>A	S6660R	p.(Ser6660Arg)	exon 80	missense	DCM	BA1	/	B		[49]
chr1-228338918-G-A	NM_001386125.1	c.20021G>A	S6674N	p.(Ser6674Asn)	exon 80	missense	DCM	BA1	/	B	MYH7 p.R1500W (not provide)	[34]
chr1-228339964-C-T	NM_001386125.1	c.20149C>T	Q6717*	p.(Gln6717Ter)	exon 81	nonsense	HCM	PM2_Supporting	1	VUS		[45]
chr1-228339970-G-A	NM_001386125.1	c.20155G>A	D6719N	p.(Asp6719Asn)	exon 81	missense	HCM	BS1, BP4, PS4_Supporting	−4	LB		[37,43]
chr1-228340049--G	NM_001386125.1	c.20234dupG	V6746Cfs*4	p.(Val6746CysfsTer4)	exon 81	frameshift	HCM	PM2_Supporting	1	VUS		[38]
chr1-228340057-G-C	NM_001386125.1	c.20242G>C	A6748P	p.(Ala6748Pro)	exon 81	missense	ARVC	BS1, PP3	−3	LB		[34]
chr1-228340746-G-A	NM_001386125.1	c.20426G>A	R6809Q	p.(Arg6809Gln)	exon 83	missense	HCM	BS1, BP4, PS4_Supporting	−4	LB	MYBPC3 p.E334K (LP) MYPN p.P1100L (VUS)	[37,43]
chr1-228341476-G-A	NM_001386125.1	c.20767G>A	D6923N	p.(Asp6923Asn)	exon 85	missense	DCM	PM2_Supporting, PS3_Supporting, BS4_Supporting	1	VUS		[31]
chr1-228359986--C	NM_052843.4	c.19098dup	G6367Rfs*87	p.(Gly6367ArgfsTer87)	exon 81	frameshift	HCM	PM2_Supporting	1	VUS		[38]
chr1-228360075-AGAG-	NM_052843.4	c.19185_19188del	R6395Sfs*44	p.(Arg6395SerfsTer44)	exon 81	frameshift	HCM	/	0	VUS		[38]
chr1-228362590-C-T	NM_001386125.1	c.21547C>T	R7183*	p.(Arg7183Ter)	exon 91	nonsense	Cardiomyopathy	/	0	VUS		[41]
chr1-228362689-A-	NM_001386125.1	c.21646delA	R7216Gfs*103	p.(Arg7216GlyfsTer103)	exon 91	frameshift	HCM	BS1	−4	LB	MYH7 p.E379K (not provide)	[38]
chr1-228365065-CT-G	NM_001386125.1	c.21797_21798delCTinsG	T7266Rfs*53	p.(Thr7266ArgfsTer53)	exon 92	frameshift	LVNC	PM2_Supporting	1	VUS		[30]
chr1-228365560-C-T	NM_001386125.1	c.21934C>T	R7312W	p.(Arg7312Trp)	exon 93	missense	LVNC	BS1	−4	LB		[40]
chr1-228365561-G-T	NM_001386125.1	c.21934+1G>T	?	p.(?)	intron 93	canonical ± 1 or 2 splice sites	HCM	/	0	VUS		[38]
chr1-228368797--TG	NM_001386125.1	c.22713_22714insTG	p.T7572*	p.(Thr7572Ter)	exon 100	nonsense	HCM	PM2_Supporting	1	VUS	MYH7 p.L957V (not provide)	[38]
chr1-228369980-G-A	NM_001386125.1	c.22877G>A	R7626H	p.(Arg7626His)	exon 102	missense	DCM	BS1	−4	LB		[34]
chr1-228370012-GT-	NM_001386125.1	c.22911_22912del	S7638Mfs*30	p.(Ser7638MetfsTer30)	exon 102	frameshift	HCM	PM2_Supporting	1	VUS		[47]
chr1-228371281-G-T	NM_001386125.1	c.23374G>T	E7792*	p.(Glu7792Ter)	exon 105	nonsense	Cardiomyopathy	PM2_Supporting	1	VUS		[41]
chr1-228371395-C-A	NM_001386125.1	c.23488C>A	L7830M	p.(Leu7830Met)	exon 105	missense	HCM	PM2_Supporting, BP4_Moderate	−1	LB		[45]
chr1-228371403--C	NM_001386125.1	c.23497dup	H7833Pfs*5	p.(His7833ProfsTer5)	exon 105	frameshift	Cardiomyopathy	PM2_Supporting	1	VUS		[41]
chr1-228371741-C-	NM_001386125.1	c.23838del	S7947Pfs*82	p.(Ser7947ProfsTer82)	exon 105	frameshift	DCM,LVNC	BA1	/	B		[30,46]
chr1-228371749-G-	NM_001386125.1	c.23848del	A7950Pfs*79	p.(Ala7950ProfsTer79)	exon 105	frameshift	DCM	/	0	VUS		[30]
chr1-228371768-A-	NM_001386125.1	c.23861del	D7954Afs*75	p.(Asp7954AlafsTer75)	exon 105	frameshift	HCM	PM2_Supporting	1	VUS		[38]
chr1-228371912-A-G	NM_001386125.1	c.24005A>G	E8002G	p.(Glu8002Gly)	exon 105	missense	HCM	PM2_Supporting, PS4_Supporting, BP4_Moderate	0	VUS	MYBPC3 p.W577* (not provide)	[45]
chr1-228372028-C-	NM_001386125.1	c.24125delC	P8042Lfs*44	p.(Pro8042LeufsTer44)	exon 105	frameshift	HCM	PM2_Supporting	1	VUS	MYH7 p.M357V (not provide)	[38]
chr1-228372114--C	NM_001386125.1	c.24210dup	C8071Lfs*43	p.(Cys8071LeufsTer43)	exon 105	frameshift	Cardiomyopathy	PM2_Supporting	1	VUS		[41]
chr1-228372725-A-C	NM_001386125.1	c.24818A>C	K8273T	p.(Lys8273Thr)	exon 105	missense	HCM	PM2_Supporting, BP4	0	VUS	RAF1 p.N553H (not provide)	[34]
chr1-228372985--C	NM_001386125.1	c.25078dup	H8360Pfs*6	p.(His8360ProfsTer6)	exon 105	frameshift	HCM	PM2_Supporting	1	VUS		[38]
chr1-228373069-G-A	NM_001386125.1	c.25162G>A	G8388S	p.(Gly8388Ser)	exon 105	missense	HCM	PS4_Supporting, BP4_Supporting	0	VUS		[45]
chr1-228374013-G-A	NM_001386125.1	c.25255+1G>A	?	p.(?)	intron 106	canonical ± 1 or 2 splice sites	Cardiomyopathy	BS1, PM4	−2	LB		[41]
chr1-228374584-G-C	NM_001386125.1	c.25367-1G>C	?	p.(?)	intron 107	canonical ± 1 or 2 splice sites	LVNC	BA1	/	B		[30]
chr1-228374603-G-A	NM_001386125.1	c.25385G>A	S8462N	p.(Ser8462Asn)	exon 108	missense	HCM	PM2_Supporting, BP4_Moderate	−1	LB	MYBPC3 p.R820Q (not provide)	[45]
chr1-228374616-CCTCA-	NM_001386125.1	c.25398_25402del	I8468Cfs*17	p.(Ile8468CysfsTer17)	exon 108	frameshift	HCM	PM2_Supporting	1	VUS		[45]
chr1-228374737-G-A	NM_001386125.1	c.25519G>A	V8507I	p.(Val8507Ile)	exon 108	missense	ARVC	BS1, BP4_Moderate	−6	LB		[34]
chr1-228375728-G-T	NM_001386125.1	c.25561G>T	D8521Y	p.(Asp8521Tyr)	exon 109	missense	HCM	PM2_Supporting, BP4_Supporting	0	VUS	MYBPC3 c.G1730A p.W577X (not provide)	[45]
chr1-228377266--G	NM_001386125.1	c.26125dup	E8709Gfs	p.(Glu8709Glyfs)	exon 112	frameshift	HCM	PM2_Supporting	1	VUS		[38]
chr1-228377500-C-G	NM_001386125.1	c.26162C>G	S8721*	p.(Ser8721Ter)	exon 113	nonsense	Cardiomyopathy	/	0	VUS		[41]
chr1-228377508-G-A	NM_001386125.1	c.26170G>A	E8724K	p.(Glu8724Lys)	exon 113	missense	DCM	BA1	/	B		[34]
chr1-228377628-G-A	NM_001386125.1	c.26290G>A	V8764I	p.(Val8764Ile)	exon 113	missense	DCM	BA1	/	B	TTN c.39893-1G>A (not provide) TTN p.K3240R (not provide)	[42]
chr1-228377994-C-T	NM_001386125.1	c.26438C>T	T8813I	p.(Thr8813Ile)	exon 114	missense	HCM	PM2_Supporting	1	VUS	MYH7 p.V606M (not provide)	[45]
chr1-228378309-G-A	NM_001386125.1	c.26489G>A	R8830H	p.(Arg8830His)	exon 115	missense	HCM	BP4_Supporting	−1	LB	MYH7 p.V606M (not provide)	[45]
chr1-228378617-G-	NM_001386125.1	c.26603delG	G8868Afs*14	p.(Gly8868AlafsTer14)	exon 116	frameshift	HCM	PM4, PM2_Supporting	3	VUS	MYH7 p.R403Q (not provide)	[38]

Abbreviations: ACMG/AMP, American College of Medical Genetics and Genomics/Association for Molecular Pathology; ARVC, arrhythmogenic right ventricular cardiomyopathy; B, benign; DCM, dilated cardiomyopathy; HCM, hypertrophic cardiomyopathy; LB, likely benign; LP, likely pathogenic; LVNC, left ventricular noncompaction; P, pathogenic; VUS, variant of uncertain significance. Note: In this table, “Cardiomyopathy” denotes cardiomyopathy not otherwise specified. Variants are described according to HGVS nomenclature and mapped to the OBSCN MANE Select transcript where applicable. Co-occurring variants indicate reported variants in established or candidate cardiomyopathy-associated genes identified in the same individual. Variant classifications were assigned using ACMG/AMP criteria and should be interpreted as evidence-based prioritization rather than definitive clinical classifications.

**Table 2:** Experimental models of OBSCN-related cardiomyopathy.

Year	Experimental Model/System	Phenotype	Key Findings	Limitations	Reference
2003	Rat/mouse TAC-induced cardiac hypertrophy model	Pressure-overload cardiac hypertrophy	The OBSCN gene cluster is rapidly upregulated in the early stages of cardiac hypertrophy, playing a key role in myofibrillar remodelling and the formation of new sarcomeres.	The study mainly focused on expression and localization analyses, lacking OBSCN loss-of-function or intervention experiments; therefore, its pathogenic mechanism and causal role have not been directly demonstrated.	[23]
2006	Obscurin knockdown zebrafish model	Myofibrillar dysplasia/cardiomyopathy-associated remodelling	OBSCN deficiency results in the disorganization of the transverse arrangement of myofibrils and abnormalities in the structure of the sarcomeres. This is accompanied by impaired cardiac development and contractile function.	The study lacked genetic rescue experiments or in-depth validation of the underlying molecular mechanisms.	[63]
2007	OBSCN variant analysis in patients with HCM	HCM	The OBSCN Arg4344Gln mutation impairs the binding of obscurin to titin and affects its localization within the Z-band, suggesting that it may be involved in the pathogenesis of HCM.	The study identified variants in only a small number of cases and lacked animal models and large-scale genetic validation.	[33]
2010	RhoGEF-specific morpholino knockdown in zebrafish embryos (OBSCN-RhoMO)	Models related to abnormal myocardial development and pathological myocardial remodelling	The absence of OBSCN-RhoGEF results in ventricular hypoplasia, impaired myocardial contractility and disrupted disc formation, suggesting that OBSCN-mediated small GTPase signaling is essential for cardiomyocyte maturation and sarcomere formation	The study is based on a zebrafish embryo model; it lacks validation in long-term mammalian disease models and does not directly elucidate the specific downstream mechanisms of the RhoA/TC10 signaling pathway.	[26]
2015	Patient-based analysis of OBSCN variants in DCM	DCM	Several potentially pathogenic OBSCN variants were identified, accompanied by reduced expression of the obscurin protein, suggesting that it may contribute to the development of DCM through haploinsufficiency.	The sample size is limited, and there is a lack of animal models and direct validation of the mechanisms involved	[31]
2020	OBSCN-ΔIg58/59 knockout mouse model	Age-dependent myocardial remodelling/arrhythmia	The absence of the OBSCN Ig58/59 domain leads to age-dependent left ventricular hypertrophy, dilation, arrhythmias and abnormalities in Ca^2+^ cycling, with marked sex differences.	The research has primarily focused on mouse models; there is currently a lack of validation in human patients and an analysis of the downstream signaling mechanisms.	[20]
2020	OBSCN-R4344Q knock-in mouse model	HCM-related arrhythmias/stress-induced myocardial remodelling	The OBSCN-R4344Q mutation causes widespread abnormalities in proteins associated with Ca^2+^ handling, ion channels, the cytoskeleton and metabolism, suggesting that it promotes the development of arrhythmias and cardiomyopathy by disrupting calcium homeostasis and electrophysiological stability.	The study utilized only a small number of homozygous mice, lacked validation in human tissue, and did not fully replicate the genetic background of human heterozygous variants	[56]
2020	OBSCN-mutant iPSC-CM model	ARVC	OBSCN variants lead to lipid droplet accumulation, disruption of the Z-band structure and enhanced L-type calcium currents, accompanied by reduced Ank1.5 expression and activation of PPARγ-related lipid synthesis pathways.	The study is based on a single-patient iPSC model and lacks gene-editing rescue experiments and in vivo functional validation.	[36]
2023	OBSCN-ΔIg58/59 gene-knockout mouse model	Age-related atrial remodelling in atrial fibrillation	The absence of the OBSCN Ig58/59 domain leads to abnormal phosphorylation of Z-disc cytoskeletal proteins, Ca^2+^ cycle regulatory proteins and various kinases, thereby promoting atrial remodelling and the development of arrhythmias	The study is primarily based on proteomic association analyses and lacks functional validation of key phosphorylation sites and signaling pathways	[21]
2025	OBSCN-ΔIg58/59 gene-knockout mouse model	Atrial cardiomyopathy/atrial fibrillation-related atrial remodelling	The absence of the OBSCN Ig58/59 domain leads to Z-disc misalignment, a reduction in the TAT network and abnormal Ca^2+^ handling, thereby inducing progressive atrial fibrillation and atrial remodelling	The study is primarily based on a male mouse model and lacks validation using human tissue samples and clinical data.	[69]
2025	OBSCN knockout mouse model	Compensatory DCM	Obscurin deficiency can lead to sarcoplasmic reticulum (SR) structural collapse, abnormal Ca^2+^ handling and increased susceptibility to arrhythmias, resulting in a compensatory dilated cardiomyopathy phenotype.	The study analyzed only 8-month-old adult mice; there is a lack of validation regarding disease progression under conditions of long-term aging and other stressors.	[66]
2025	OBSCN/OBSL1 double knockout (dKO) mice	Diastolic dysfunction and impaired cardiac relaxation	Combined cardiac-specific loss of Obsc and Obsl1 caused impaired cardiac relaxation, disrupted sarcoplasmic reticulum architecture and calcium cycling, mitochondrial abnormalities, altered metabolism, and deregulated mitophagy, supporting partly redundant functions of the two proteins in the heart.	Cardiac physiological analyses were performed predominantly in male mice, limiting assessment of sex differences. Combined gene deletion does not directly establish the pathogenicity of individual human OBSCN variants.	[67]

HCM, hypertrophic cardiomyopathy; DCM, dilated cardiomyopathy; TAC, transverse aortic constriction; ARVC, arrhythmogenic right ventricular cardiomyopathy.

## Data Availability

No new datasets were generated during the preparation of this review. The data and information discussed in this article were obtained from publicly available sources, including PubMed and the Leiden Open Variation Database (LOVD), as cited in the manuscript. Any additional extracted or summarized information is available within the article and its Supplementary Materials, where applicable.

## Supplementary Materials

The following supporting information can be downloaded at: https://www.mdpi.com/article/10.3390/jcdd13100530/s1, Supplementary File S1: Methods for research-based assessment and compilation of OBSCN variants; Figure S1. Workflow for the identification and compilation of OBSCN variants reported in patients with cardiomyopathy; Table S1. Evidence strengths and points used in the research-based assessment; Table S2. Net score and final research-based classification; Table S3. Application of individual evidence criteria.
