# Supplementary material for: The Role of the OBSCN Gene in Cardiomyopathy: A Review of Molecular Mechanisms and Clinical Significance
Source: J Cardiovasc Dev Dis. 2026 Oct 21;13(10):530. doi: 10.3390/jcdd13100530 (PMC13705083; doi:10.3390/jcdd13100530)
Supplement: Supplementary file 1 [file jcdd-13-00530-s001.zip › Supplementary_File_S1.pdf]

Supplementary File S1: Methods for research-based assessment and compilation of OBSCN variants

Research-based application of ACMG/AMP criteria to OBSCN variants

ClinGen has classified the gene–disease validity of OBSCN for both hypertrophic cardiomyopathy (HCM) and dilated cardiomyopathy (DCM) as Limited; its HCM curation also notes that the disease mechanism is unknown.[1,2] We performed a research-based evaluation using the 2015 ACMG/AMP guidelines, the Bayesian point system of Tavtigian et al. (2020), ClinGen Sequence Variant Interpretation (SVI) recommendations, and relevant Cardiomyopathy Variant Curation Expert Panel (VCEP) specifications.[1–8] PVS1 was not applied at any strength; final classifications in the pathogenic direction were capped at variant of uncertain significance (VUS), whereas benign-direction evidence was assessed where applicable.[9,10] Truncating variants were considered for PM4 only when they were predicted to escape nonsense-mediated decay (NMD) and to alter protein length. These study-specific rules organize evidence and support descriptive mapping; they are not an approved OBSCN-specific VCEP specification and do not replace clinical laboratory classifications.

Except for two variants that could not be mapped to MANE Select, variants were named and mapped using MANE Select transcript NM_001386125.1 and protein NP_001373054.1 (116 exons; predicted protein length, 8,925 amino acids). Population data were taken from gnomAD v4.1.1. BA1 and BS1 were assessed using the GroupMax Filtering AF (95% confidence; FAF95, a one-sided 95% lower confidence bound) for the selected dataset, with its corresponding ancestry group recorded. GroupMax identifies the group with the highest FAF, not necessarily that with the highest observed AF. PM2 was assessed separately from site-specific allele counts (AC) and callable allele numbers (AN), using the one-sided 95% upper confidence bound calculated with the Confit-de-MAF tool recommended in the Cardiomyopathy VCEP specification. The ancestry groups and frequency statistics underlying the two types of evidence were recorded separately. REVEL was prespecified for missense prediction and SpliceAI for splice prediction.

The allele-frequency thresholds corresponding to PM2, BA1, and BS1 were prespecified with reference to the ClinGen Cardiomyopathy VCEP specifications to facilitate exploratory, research-oriented stratification of OBSCN variants, rather than formal clinical variant classification. Thus, frequency-dependent B/LB/VUS calls and any changes in inclusion in the mapping figure should be reported as limitations. We did not assess classification sensitivity to alternative frequency thresholds. The effect of threshold selection on B/LB classifications and inclusion in the mapping analysis therefore remains uncertain..

Applicable, nonduplicated evidence was summed using the point system of Tavtigian et al. (2020), with final classifications assigned according to Tables S1 and S2. BA1 was applied as a stand-alone benign criterion. Criteria-specific application rules are detailed in Table S3.

Literature search and database screening

We collected OBSCN variants reported in patients with cardiomyopathy from PubMed, Europe PMC, and the Leiden Open Variation Database (LOVD). Literature source identification was verified by searches of PubMed and Europe PMC on 1 September 2026. Searches covered publications from database inception through 1 September 2026, inclusive, without language restrictions.

PubMed was searched using a gene-name-only query across all indexed fields to identify studies that might not mention cardiomyopathy in their titles or abstracts:

(OBSCN[All Fields] OR obscurin[All Fields]) AND ("1800/01/01"[Date - Publication] : "2026/09/01"[Date - Publication])

A complementary Europe PMC query combined the gene names with cardiomyopathy terms and abbreviations:

(OBSCN OR obscurin) AND (cardiomyopath* OR HCM OR DCM OR ARVC OR ARVD OR LVNC OR noncompaction OR "non-compaction") AND FIRST_PDATE:[* TO 2026-09-01]

Europe PMC was searched without publication-type restrictions, and its default search scope included available indexed full text. The PubMed and Europe PMC searches returned 300 and 557 records, respectively.

Reference lists of relevant reviews retrieved by the database searches were examined to identify additional eligible studies. Patient-level variant data were extracted from the original studies rather than the reviews.

Eligible studies were case reports, case series, and cohorts providing specific OBSCN variant information for patients with a documented cardiomyopathy diagnosis. Reviews, studies limited to functional or animal experiments, and studies without relevant patient variant data were excluded as patient-data sources. Patient data were extracted from eligible studies even if those studies also included functional experiments. Variant pathogenicity was not an eligibility requirement. Cardiomyopathy phenotypes followed the original study diagnoses; the included phenotype categories were HCM, DCM, ARVC, LVNC, and cardiomyopathy not otherwise specified. Heart failure, arrhythmia, or sudden death without an explicitly documented cardiomyopathy diagnosis did not independently meet the phenotype criterion.

LOVD OBSCN individual records were screened on 1 September 2026 for an explicitly documented cardiomyopathy phenotype, and variant, phenotype, and source information was extracted. The same phenotype criterion was applied to the LOVD records. Of the 21 individual records screened, five met this criterion, whereas the remaining 16 were excluded. All five eligible records overlapped with the PubMed-derived data and were not counted as additional independent observations.

The final compilation comprised 21 studies and five eligible LOVD records, all of which overlapped with the literature-derived data. Of the 21 studies, 19 were retrieved directly by the database searches and two were identified through reference checking. After merging and deduplication, the compilation contained 138 distinct variants (Figure S1).

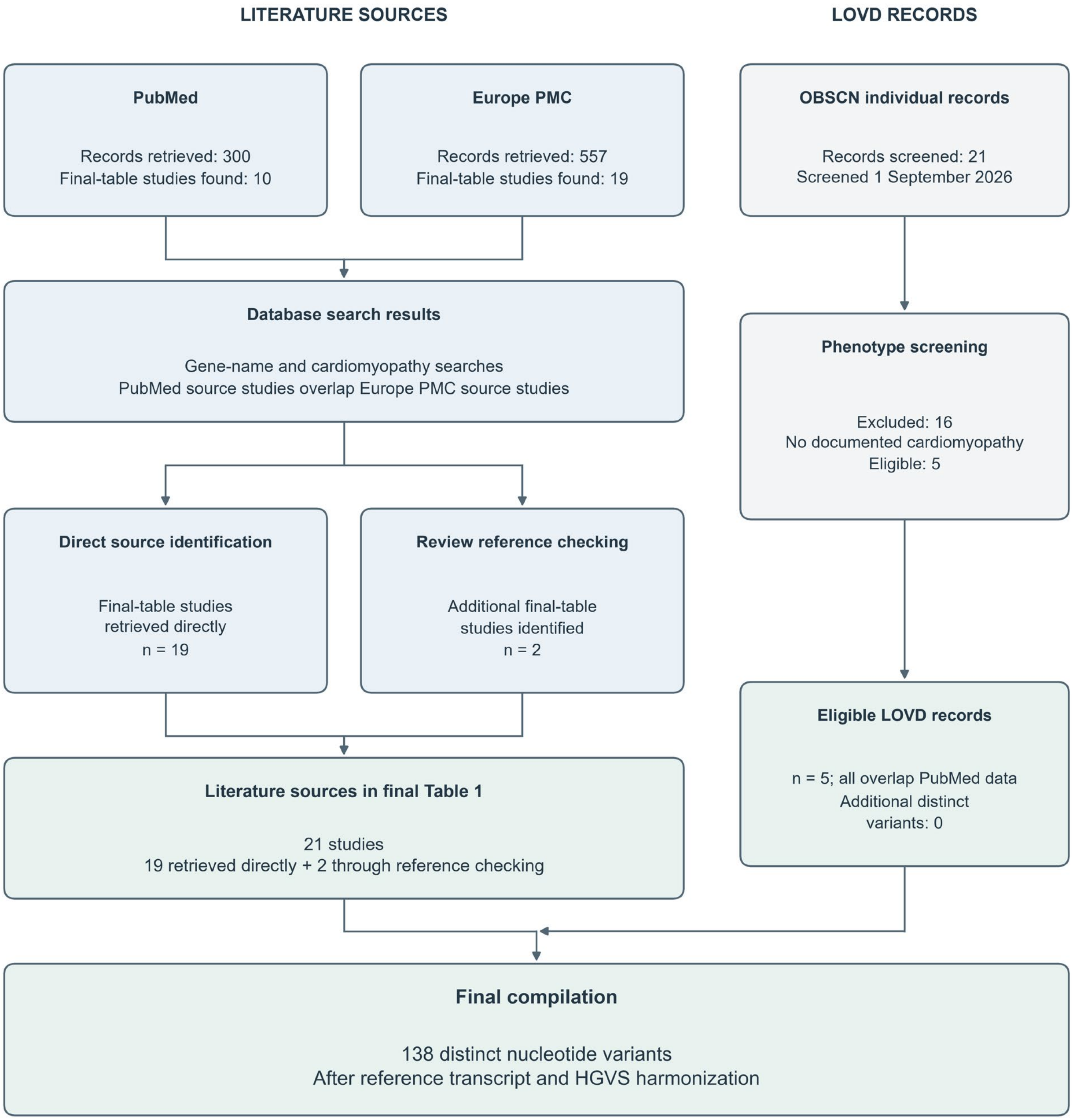

Figure S1. Workflow for the identification and compilation of OBSCN variants reported in patients with cardiomyopathy.

Handling of duplicate records

The unit of compilation was a distinct nucleotide variant. The same OBSCN variant reported in different patients, studies, or LOVD records was combined into one variant entry while retaining sources, phenotypes, and evidence. Duplicates were identified after harmonization of the reference transcript and HGVS descriptions by nucleotide change and position.[3] Distinct nucleotide changes were not combined even when they produced the same protein alteration. Records whose equivalence or nomenclature could not be established reliably were reviewed separately. Merging variant records did not imply that carriers were the

same individual; confirmed repeat reports of one patient or family were counted only once as case-level evidence, including when both literature and LOVD contained the record.

Data extraction and evidence assessment

For each distinct variant, we recorded original nomenclature, reference sequence, normalized nucleotide and protein changes, variant type, cardiomyopathy phenotype, literature or LOVD source, and available family, functional, and co-occurring variant data. Original author or database classifications were kept separate from this study's research-based classification. Discordant source descriptions were retained for verification; information that could not be confirmed was marked missing rather than inferred.

Population data recorded the gnomAD release, access date, selected dataset (exomes, genomes, or joint), site quality and coverage, and available ancestry-specific AC, AN, and AF. For BA1 and BS1, the GroupMax FAF95 and its ancestry group were recorded. For PM2, AC, AN, and calculated upper bounds were recorded for the five principal groups, along with carriers in other groups and the rationale for adjudication. The GroupMax FAF group was not assumed to be the highest-observed-AF group or the sole PM2 evaluation group. AC and AN from distinct datasets were not added without verifying non-overlap. Database absence or unavailable prediction scores did not imply biological absence or a benign consequence. Each criterion was recorded with code, strength, points, and source evidence; unverifiable or inapplicable evidence was not scored. Conflicts among database labels, predictions, and functional or family data were retained.

NMD status was predicted from the position of the new termination codon relative to the last exon–exon junction in NM_001386125.1, using the approximate 50–55-nucleotide rule. A new stop codon in the last exon or within 50 nucleotides upstream of the final junction was labeled as predicted to escape NMD; one more than 55 nucleotides upstream was labeled as predicted to trigger NMD. Stops in the 50–55-nucleotide boundary region, uncertain transcript consequences, or relevant exceptions were labeled indeterminate. Frameshifts were assessed using the new stop codon rather than the insertion/deletion site. Splice variants were not assigned an NMD outcome solely from splice-site position without evidence of transcript consequences. These are predictions, not experimental demonstrations of transcript or protein effects.[10]

The two variants that could not be mapped to MANE Select were retained in the compilation with their original descriptions, sources, and available evidence. Their exon or protein positions were not inferred, and they were excluded from coordinate-dependent mapping statistics. Evidence dependent on unverified transcript consequences or NMD was not scored.[9,10]

Variant mapping and descriptive analysis

All distinct variants were retained in the compilation. The main positional figure excluded research-classified B and LB variants and displayed the remaining mappable VUS variants, including those with a net score of zero, annotated by variant type and evidence. We described the distribution of compiled variants across exons and protein domains without testing for enrichment. Locally higher counts were not interpreted as pathogenic hot spots or used to assign PM1 or PS4. The distribution is affected by literature selection, testing scope, sequencing coverage, and repeated reporting; it cannot estimate population disease risk or establish an OBSCN disease mechanism.

References

1. Clinical Genome Resource (ClinGen) OBSCN - Dilated Cardiomyopathy: ClinGen Gene-Disease Validity Submission SGC-105648.4 Available online: https://thegencc.org/submissions/SGC-105648.4.
2. Clinical Genome Resource (ClinGen) OBSCN - Hypertrophic Cardiomyopathy: ClinGen Gene-Disease Validity Submission SGC-105256.3 Available online: https://thegencc.org/submissions/SGC-105256.3.

3. Richards, S.; Aziz, N.; Bale, S.; Bick, D.; Das, S.; Gastier-Foster, J.; Grody, W.W.; Hegde, M.; Lyon, E.; Spector, E.; et al. Standards and Guidelines for the Interpretation of Sequence Variants: A Joint Consensus Recommendation of the American College of Medical Genetics and Genomics and the Association for Molecular Pathology. *Genet. Med.* **2015**, *17*, 405–424, doi:10.1038/gim.2015.30.
4. ClinGen Cardiomyopathy Variant Curation Expert Panel *ClinGen Cardiomyopathy VCEP Criteria Specification for TNNI3 (GN098)*; Clinical Genome Resource (ClinGen);
5. ClinGen Cardiomyopathy Variant Curation Expert Panel *ClinGen Cardiomyopathy VCEP Criteria Specification for MYH7 (GN002)*; Clinical Genome Resource (ClinGen);
6. ClinGen Cardiomyopathy Variant Curation Expert Panel *ClinGen Cardiomyopathy VCEP Criteria Specification for MYBPC3 (GN095)*; Clinical Genome Resource (ClinGen);
7. Kelly, M.A.; Caleshu, C.; Morales, A.; Buchan, J.; Wolf, Z.; Harrison, S.M.; Cook, S.; Dillon, M.W.; Garcia, J.; Haverfield, E.; et al. Adaptation and Validation of the ACMG/AMP Variant Classification Framework for MYH7-Associated Inherited Cardiomyopathies: Recommendations by ClinGen's Inherited Cardiomyopathy Expert Panel. *Genet. Med.: Off. J. Am. Coll. Med. Genet.* **2018**, *20*, 351–359, doi:10.1038/gim.2017.218.
8. Tavtigian, S.V.; Harrison, S.M.; Boucher, K.M.; Biesecker, L.G. Fitting a Naturally Scaled Point System to the ACMG/AMP Variant Classification Guidelines. *Hum. Mutat.* **2020**, *41*, 1734–1737, doi:10.1002/humu.24088.
9. Bean, L.J.H.; Funke, B.; Carlston, C.M.; Gannon, J.L.; Kantarci, S.; Krock, B.L.; Zhang, S.; Bayrak-Toydemir, P. Diagnostic Gene Sequencing Panels: From Design to Report-a Technical Standard of the American College of Medical Genetics and Genomics (ACMG). *Genet Med* **2020**, *22*, 453–461, doi:10.1038/s41436-019-0666-z.
10. Abou Tayoun, A.N.; Pesaran, T.; DiStefano, M.T.; Oza, A.; Rehm, H.L.; Biesecker, L.G.; Harrison, S.M. Recommendations for Interpreting the Loss of Function PVS1 ACMG/AMP Variant Criterion. *Hum Mutat* **2018**, *39*, 1517–1524, doi:10.1002/humu.23626.
